# Large potassium shifts during dialysis enhance cardiac repolarization instability

**DOI:** 10.1007/s40620-020-00880-4

**Published:** 2020-10-15

**Authors:** Dominik Schüttler, Ulf Schönermarck, Felix Wenner, Marcell Toepfer, Konstantinos D. Rizas, Axel Bauer, Stefan Brunner, Wolfgang Hamm

**Affiliations:** 1 Medizinische Klinik Und Poliklinik I, Campus Grosshadern and Innenstadt, University Hospital Munich, Ludwig-Maximilians University Munich (LMU), Marchioninistraße 15, 81377 Munich, Germany; 2 DZHK (German Centre for Cardiovascular Research), Munich Heart Alliance (MHA), Partner Site Munich, Munich, Germany; 3 Walter Brendel Centre of Experimental Medicine, Ludwig-Maximilians University Munich (LMU), Munich, Germany; 4 Nephrology Division, Medizinische Klinik Und Poliklinik IV, Klinikum der Universität München - Campus Großhadern, Munich, Germany; 5 Dialysis Center Murnau, Murnau, Germany; 6 University Hospital for Internal Medicine III, Medical University Innsbruck, Innsbruck, Austria

**Keywords:** Autonomic dysfunction, Periodic repolarization dynamics, Dialysis, Potassium shift, Arrhythmia

## Abstract

**Background:**

Patients with end-stage kidney disease are at high risk for the development of arrhythmias and sudden cardiac death (SCD). This has been especially attributed to large potassium shifts during hemodialysis (HD), and malignant arrhythmias are closely linked to dysfunction of the autonomic nervous system. Nevertheless, there is still a lack of methods for risk stratification in these patients.

**Methods:**

In the present pilot study we investigated changes of the novel ECG-based biomarker periodic repolarization dynamics (PRD) mirroring the effect of efferent sympathetic nervous activity on the ventricular myocardium in 18 patients undergoing routine hemodialysis. High-resolution ECGs were recorded throughout the dialysis and PRD values were calculated out of 30 min intervals at the start and the end of dialysis.

**Results:**

We detected a clear correlation between the intradialytic potassium shift and the increase in PRD levels (Spearman correlation coefficient *R* = 0.62, *p* = 0.006). Patients with a potassium shift > 1 mmol/l showed significantly increased levels of PRD at the end of dialysis when compared to patients with potassium shifts ≤ 1.0 mmol/l [delta PRD 2.82 (IQR 2.13) vs. − 2.08 (IQR 3.60), *p* = 0.006]. Spearman analysis showed no significant correlation between PRD changes and fluid removal (*R* = − 0.23, *p* = 0.36).

**Conclusions:**

We provide evidence that large potassium shifts during HD enhance sympathetic activity-associated repolarization instability. This could facilitate the occurrence of malignant arrhythmias, and PRD measurements might serve as a non-invasive monitoring tool in HD patients in future.

## Introduction

Cardiovascular and renal diseases often coexist, and both contribute to a substantially high morbidity and mortality. Hemodialysis (HD) patients suffer from an estimated 14-fold increased mortality due to sudden cardiac death (SCD) compared to patients with normal kidney function. About one third of deaths in patients with end-stage kidney disease are related to SCD, mostly caused by malignant cardiac arrhythmias [1].

Aggressive removal of potassium and fluids resulting in large serum potassium shifts has been demonstrated to significantly increase risks for sudden cardiac death and arrhythmias in HD patients [2]. Furthermore, deaths from cardiac arrests are more common after the long 2-day inter-dialytic break, possibly due to larger electrolyte shifts especially of potassium [2]. A study investigating HD patients with implantable cardioverter defibrillators (ICDs) showed that the magnitude of arrhythmias occurred during dialysis sessions [3]. The use of dialysate with constant and low potassium concentrations increases the risk for arrhythmias during HD compared to potassium profiled dialysate with smoother potassium removal [4]. Consequently, one of the main goals is to maintain serum potassium during intradialytic and interdialytic intervals within a narrow normal range, but the prognostic impact of rapid alterations of potassium levels during HD is still insufficiently explored [5]. In addition to tachyarrhythmias a large proportion of arrhythmias consist of asystole and bradyarrhythmias [6].

Nevertheless, the pathophysiological background of the occurrence of SCD in patients with end-stage kidney disease remains complex and is insufficiently investigated. An improved risk stratification with clinically relevant biomarkers is urgently needed [7].

As the contribution of autonomic nervous system (ANS) dysfunction to the development of malignant arrhythmias is well known, we hypothesized that potassium shifts during routine HD might lead to an increased susceptibility to sympathetic activity-associated repolarization instability. Periodic repolarization dynamics (PRD) is a novel ECG-based biomarker which mirrors the effect of efferent sympathetic activity on the level of the ventricular myocardium [8]. Calculation of PRD is based on the quantification of low-frequency oscillations (≤ 0.1 Hz) of cardiac repolarization. First, the angle between successive repolarization vectors (*dT*°) is determined and plotted over time. Then low-frequency components are assessed using wavelet analysis [8]. Increased PRD levels have been shown to be a strong predictor of mortality in patients with ischemic and non-ischemic cardiomyopathy [8, 9].

## Methods

**Fig. 1:**
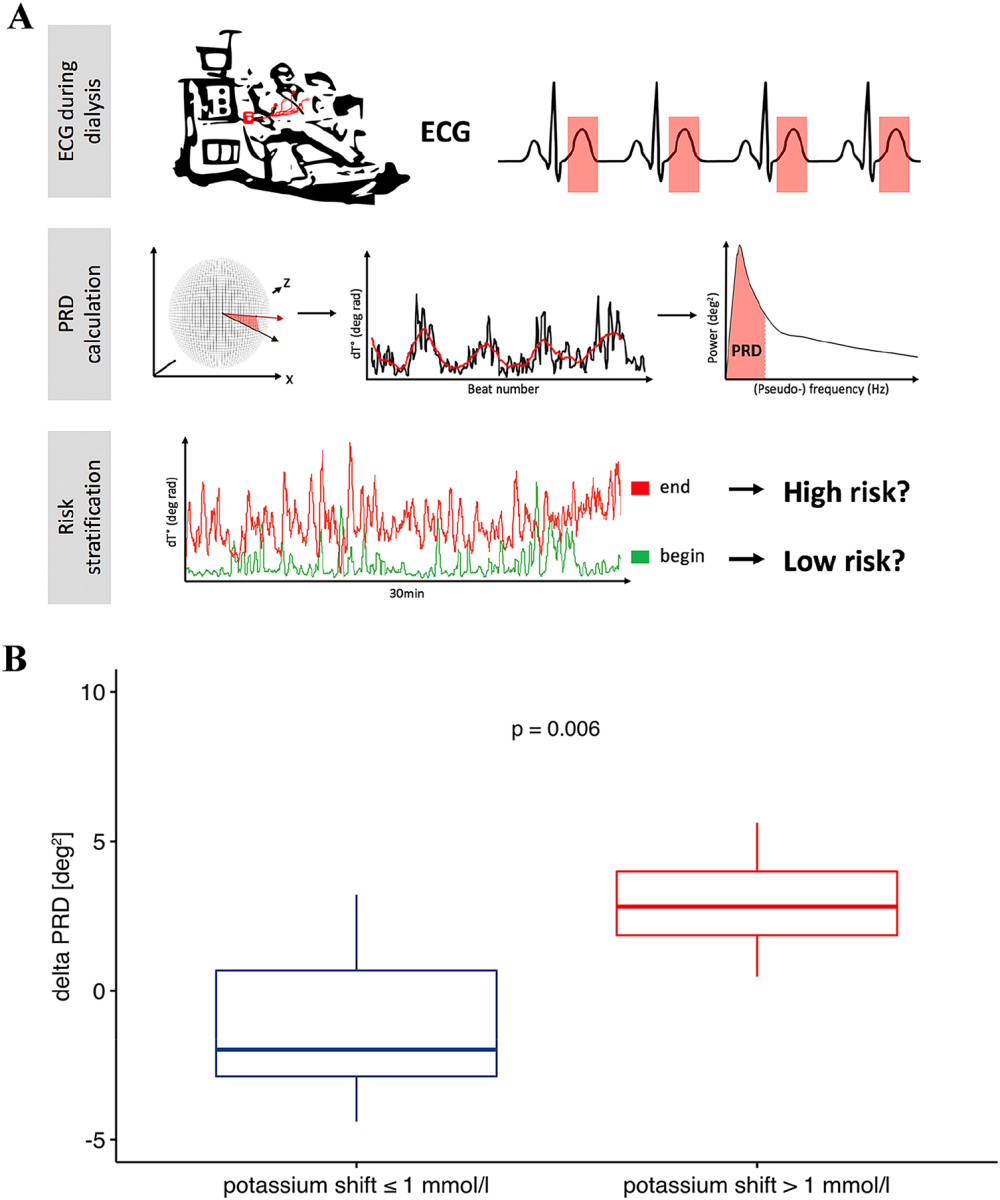

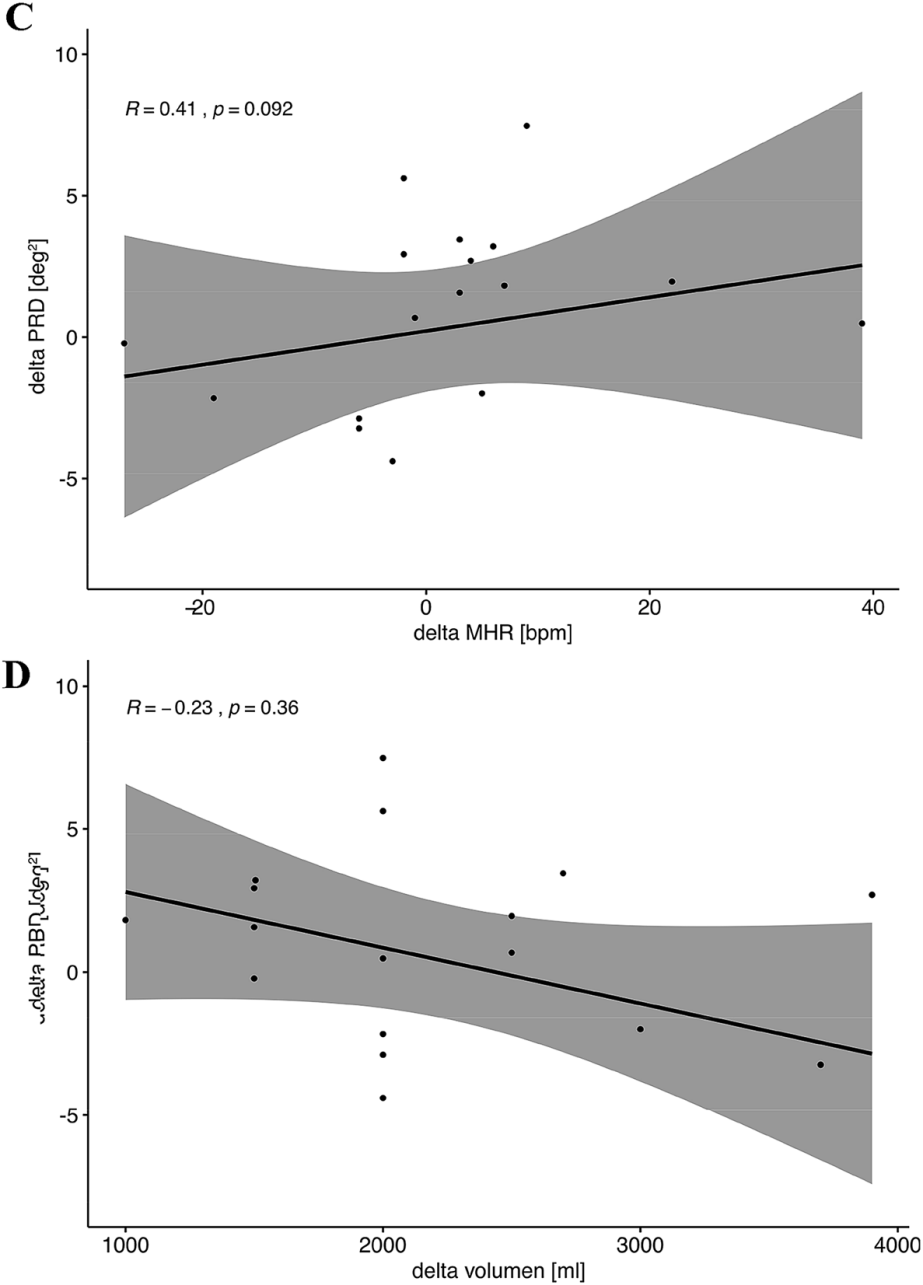
High resolution ECG (1000 Hz) was recorded during the entire dialysis in orthogonal (Frank)-lead configuration. Spatiotemporal information of each T wave is integrated into a single T-wave vector. PRD is calculated via beat-to-beat changes of the T-wave vector (dT°) with periodic components of repolarization in the low-frequency range (≤ 0.1 Hz). Illustration of low dT° signal at the beginning of dialysis and high dT° signal with characteristic low-frequency oscillation at the end of dialysis in a patient with a high potassium shift assuming increased risk due to increased cardiac repolarization instability (**a**). Altered PRD levels at the end of dialysis dependent on the amount of potassium shift. (Mann–Whitney-Wilcoxon-test, *p* < 0.05 estimated as statistically significant) (**b**). Spearman correlation analysis showing association between differences in PRD levels and corresponding heart rate changes (“delta” refers to differences of values between end and start of dialysis) (**c**). Spearman correlation analysis showing association between differences in PRD levels and fluid removal (“delta” refers to differences of values between end and start of dialysis) (**d**)

In the present study we included 18 patients undergoing routine hemodialysis (12 male, 6 female, mean age 65.1 yr. ± 13.5 yr.). Six subjects had known coronary artery disease, four peripheral artery disease, four diabetes mellitus and 11 hypertension. Electrolytes were quantified via blood gas analysis before and after hemodialysis. During the entire dialysis time we tracked the spatiotemporal properties of cardiac repolarization on a beat-to-beat basis via high-resolution ECG (Schiller medilog AR4 plus, 1000 Hz, Schiller Medizintechnik, CH) in orthogonal Frank-lead configuration. Figure 1a shows a schematic illustration of the experimental setting. ECG-signals were analyzed using Matlab with established algorithms for the calculation of PRD. Standardized ECG filter settings (high-pass 0.1 Hz; low-pass 100 Hz) were used. The spatiotemporal information of each T wave was integrated in a vector dT°. The instantaneous degree of repolarization instability was subsequently measured by means of the angle *dT°* between two successive T° vectors and plotted over time [8]. Afterwards, PRD was calculated by the use of wavelet analysis in the low frequency spectrum (≤ 0.1 Hz). Here, we computed PRD levels in each study participant out of 30 min ECG intervals at the beginning and the end of the HD session. PRD calculation was performed as previously described [8]. Figure 1a visualizes the principle of dT° assessment and the calculation of PRD.

Mann–Whitney-Wilcoxon test was used to reveal statistical differences in PRD changes dependent on potassium removal (> 1 mmol/l vs. ≤ 1 mmol/l) and to show differences between heart rates at the end and the beginning of the dialysis session. Spearman analysis was used to test for a correlation between changes in PRD and potassium levels, between changes in PRD and heart rate as well as between changes in PRD and fluid removal. To further determine the association between PRD and changes in heart rate we performed linear regression analysis (dependent variable: PRD at the end of dialysis; independent variables: PRD at beginning of dialysis and differences in heart rate at the end and beginning of dialysis).

## Results

Mean fluid removal was 2270 ml ± 820 ml during an average HD time of 4 h 3 min. Mean potassium at baseline was 4.75 ± 0.74 mmol/l and 3.87 ± 0.32 mmol/l after dialysis. Mean heart rate did not change significantly when comparing the first 30 min and the last 30 min of each dialysis session [74.5 bpm (IQR 11.5 bpm) vs. 79 bpm (IQR 14.5 bpm) *p* = 0.590; Mann–Whitney-Wilcoxon test].

When we investigated the changes in PRD levels between the start and the end of the dialysis session, we interestingly did not detect relevant differences in all patients undergoing HD. However, we found a significant correlation between the potassium shift and the increase of PRD levels (Spearman correlation coefficient *R* = 0.62, *p* = 0.006). Those patients with a potassium shift > 1 mmol/l showed significantly increased levels of PRD at the end of dialysis when compared to patients with potassium shifts ≤ 1.0 mmol/l (delta PRD 2.82 (IQR 2.13) vs. − 2.08 (IQR 3.60), *p* = 0.006, Mann–Whitney-Wilcoxon test, Fig. 1b). Figure 1a includes an exemplary tracing of a dT° signal (which is the basis of the PRD calculation) showing low signals at the beginning and elevated signals with the typical low frequency oscillations at the end of HD in a patient with a high potassium shift. Of note, alterations in PRD levels showed no significant correlation with changes in heart rates (Spearman correlation coefficient *R* = 0.41, *p* = 0.092, Fig. 1c). Additionally, there was no correlation between changes in PRD levels and fluid removal (Spearman correlation coefficient *R* = − 0.23, *p* = 0.36, Fig. 1d). Linear regression analysis revealed that changes in PRD levels were independent of changes in heart rate (*p* = 0.27).

## Discussion

In summary, we observed a link between potassium shifts during HD and PRD levels which indicates that removal of large amounts of potassium enhances the effect of efferent sympathetic activity on the ventricular myocardium. These changes were shown to be independent of changes in heart rates, a fact that has already been demonstrated in previous studies [8, 10]. The independence of heart rate is important since fluid removal during HD per se might alter heart rate.

Our study could be limited by the fact that 12 subjects received betablockers, which are known to attenuate sympathetic activity. Additionally, four subjects had diabetes mellitus which might result in dysautonomia. Nevertheless, a large proportion of study participants in previous trials investigating PRD were under optimal medical therapy including betablockers and many had diabetes mellitus [8, 9]. PRD showed its prognostic power despite this fact in those studies. Additionally, our study might be limited as we did not detect any arrhythmias in our ECG recordings and a large proportion of arrhythmias attributed to HD-related SCD consist of asystole and bradyarrhythmias [6]. Thus, the clinical significance of the alterations in sympathetic activity-mediated repolarization instability due to potassium shifts as detected by us has to be evaluated in future prospective trials. Nevertheless, our pilot study demonstrates a physiological link between changes of potassium shift and vulnerability of the heart to sympathetic activity-associated repolarization instability. This could, at least in part explain the increases in malignant arrhythmias described in literature. Further studies should investigate the role of PRD for risk stratification in patients undergoing hemodialysis. Continuous monitoring of PRD during HD could be used as an instantaneous feedback mechanism on HD parameters in order to reduce the risk of potentially life-threatening arrhythmias caused by potassium shifts.
